# New York Correspondence

**Published:** 1859-09

**Authors:** D. C. O’Keefe

**Affiliations:** New York


### NEW YORK CORRESPONDENCE.

New York, September 1, 1859.

*Messrs. Editors:*—It is estimated that there are in this city 1800 regular Physicians, 120 Hahnemanneans, and how many *non-de-scripts* the record sayeth not. The period of high professional position here is comparatively brief—ten or twenty years being spent in reaching it. Considering, therefore, the number of medical men who die within twenty years of active professional life, it will be seen that the successful aspirants must be comparatively few.

Let us suppose a young man of good elementary education commencing the study of medicine, with the view of making this city the field of his professional labors; he enters the office of a physician, for which he pays one hundred dollars per annum; attends Lectures three or four years, and is admitted to his degree. His next step is to obtain the position of *Interne* in one of the principal Hospitals, and here serves a term of two years. He next visits the Capitols of Europe, and spends two or three years at the feet of their medical Gamaliel's. He is now prepared to commence the practice of his profession, and if he have ordinary talents the road to distinction, though still rugged, is open. If he have wealth and good social position, his progress will be rapid and his ascent easy, but let him be obscure, oppressed by poverty, and the burdens of a family, to gain the position to which his talents and acquirements entitle him, *hic labor, hoc opus est.* But, whether rich or poor, he has unfurled his sails to the breeze, and selected the particular course in which his barque is to steer—for it must be borne in mind that every medical man in this city is more or less of a speciality, and here devotes many a weary hour to practice, profitable only in the experience it furnishes.

These Institutions are often served by the first medical men in the city, without any pecuniary compensation whatever, so that much time and labor is devoted merely to the acquisition of experience. The Dispensaries serve as agencies for gaining access to the masses, and the position of Hospital Physician or Surgeon is supposed to promote the reputation of its occupant. Many young men, moreover, attach themselves to the Colleges, "grinding and quizzing" in connection with the lectures, and thus secure a fair income from private instruction.

It is thus, after a long and hard struggle of nearly half a life-time that a lucrative professional position is obtained, and the period of its fruition, as stated, is comparatively brief.

But the man who has passed through this ordeal, is an experienced practitioner and accomplished physician. He has enjoyed opportunities of culture inaccessible to the great majority of medical men ; he devotes all his energies to a class of diseases, and he must be stupid indeed if he do not become proficient in his department. The result is that the Physicians and Surgeons of this city, whether connected with Colleges or not, are eminently qualified for the discharge of their duties; and reflect honor on our profession. It is no uncommon occurrence to hear these men use two or three foreign languages in going through the wards of a hospital, and this is no idle or unnecessary accomplishment, for New York, in its hospital population, is as much a foreign as an American city.

Another important advantage the profession here enjoy is the frequent opportunities afforded of studying pathology on the cadaver. Besides the numerous autopsies made at public institutions, they are also of frequent occurrence in private practice. According to my observation, which of course is limited, post-mortem examinations are much more frequently allowed and performed than at the South, where such privileges, in the case of whites at least, are scarcely thought of.

The best evidence of a correct public opinion on this subject, in this State, is the legalizing of anatomical dissections, it being one of the few States in the Union which has shown a proper appreciation of human dissection. Its legal action on this subject, however, is not free from inconsistency ; for, whilst practical anatomy is permitted by statute, the procuring of the material for prosecuting it is a punishable offence. Still a valuable concession has been obtained, and, in my opinion, the profession has itself to thank for it; by the anxiety evinced for making autopsies in public and private practice, the people become accustomed to them and convinced of their importance. May not the tardiness of other States, in this regard, be owing to the apathy of the profession within their limits on the subject of pathological anatomy? I believe the profession at the South is to blame in this respect; it is notoriously negligent in the performance of autopsies, and that too in the midst of abundant opportunities. Whatever obstacles exist in regard to whites, there are none in the case of blacks. I have never been refused a post-mortem of a negro, and presume my case is not singular. The truth is, physicians do not seek them, and when *requested* to perform them insist on the absurd usage of charging a fee for service that should be voluntarily rendered in every practicable case. The cause of this indifference, (to judge by personal experience, the usual standard of comparison,) I take to be an inability to appreciate pathological changes when they exist, and therefore the examination is barren of useful results. The physician feels, after the unfavorable termination of a case that he has performed his duty, and can do no more; here, on the contrary, the medical attendant is not satisfied that he has discharged all the obligations resting on him, until he has penetrated the veil which concealed the disease from his vision, and the public participate, to some extent, in this feeling. How appropriate it would be for the Empire State of the South, after the example of her Northern representative, to take the lead of her sister States in legalizing anatomical dissections, and thus contribute an important element to the relief of human suffering.

Against a profession thus fortified by a high order of talents and extensive acquirements, you will be prepared to hear that the monstrosity, yclept Homœopathy, has made little progress, and that little owing not to its inherent merits, but a virtual abandonment of its peculiar doctrines. Its disciples, with few exceptions, are men of small calibre, who would inevitably fail in a fair competition in the regular profession, and who use its mysteries as a prop for their weakness. The exceptions are regularly educated physicians, of clever parts, who adopt the name and the *similia similibus* portion of the system but utterly reject the infinitessimal dilutions. To judge by the gasconade of the novitiates of this system who find their way into the country, one would suppose that its merits were so transcendantly overpowering that its rapid progress would be irresistible; but here, where it has had a fair field for nearly half a century, it is absolutely declining. There is not a public institution of any kind—hospital, infirmary, or dispensary—under its control; and this, of course, is a fair criterion of its influence in the community—these institutions being under the direction and management of the people. The same may be said of it in Great Britain and the Continent of Europe, where it originated. Its incursions into the country, therefore, (your own flourishing young city for example, where I should think the intelligence of the profession would allow it but a precarious existence,) are not a proof of its extension, but an illustration of the well-known fact that when impostures are detected, exposed, and moribund in one quarter, they will struggle for life in another.

Any one desirous of knowing the height, and depth, and extent of the absurdities of this system, should read the "Organon" of its illustrious founder, and see what an inveterate old dreamer he was. Laugh over his chronic miasms—syphilis, sycosis and scabies—the "fundamental" cause of all chronic maladies. Had he stated syphilis to be the cause of much chronic and acute vice in the system, one could agree with him, nor need his opinion that it is never entirely eradicated, be gainsayed. But sycosis, the harmless, though obstinate Barber's itch, with its cryptogams and sporules, I know to be consistent with perfect health; and scabies, with its acarus, his great chronic Pandora's box of the human race, is purely a local disease, and completely amenable to the sulphur ointment. At the risk of being tedious, and to show the extent of the whims of the father of Homœpathy, I will enumerate the chronic diseases, which, in his opinion, were caused by the itch. It is, says he, "the sole true and fundamental cause that produces all the other countless forms of disease, which, under the names of nervous debility, hysteria, hemicrania, hypochondriasis, insanity, melancholy, idiocy, madness, epilepsy, and spasms of all kinds, softening of the bones, or rickets, scoliosis and cyphosis, caries, cancer, fungus hœmatodes, pseudo-morphæ of all kinds, gravel, gout, hœmorrhoids, jaundice and cyanosis, dropsy, amenorrhea, gastroragia, epitaxis, hemoptysis, hematuria, metroragia, asthma, phthisis ulcerosa, impotency and sterility, deafness, cataract and amaurosis, paralysis, loss of sense, pains of every kind, &c., appear in our pathology as so many peculiar, distinct and independent diseases." Verily, O acarus scabei! thou art a peccant animalcule, and our German philosopher deserves the thanks of posterity in discovering thy depredations, even at the expense of twelve years of study and research. Such was the manner of man who originated a system which claims merits tantamount to a divine revelation, and has no stronger recommendation than its incomprehensible absurdities.

Another doctrine equally audacious, and still literally followed by his disciples, is that the "*tout ensemble*" of the symptoms constitute the disease itself—that the physician need not go beyond the external manifestations of the disease, and therefore anatomy, physiology and pathology must be superfluous.

Homœopaths of the present day, if honest, act strictly on this theory, and prescribe only for symptoms, regardless of their origin. Thus, if vomiting be the external manifestation, it is treated as such, whether it be owing to irritant substances in the stomach, pregnancy, dyspepsia or cancer. To my mind the system is wrong and absurd in all its parts; but in none so glaringly as this. Were such a view of disease correct, it would save our profession much time and labor; the patient investigations in chemical and microscopic pathology, were time mis-spent; the minute observations of the immortal Lœnnec, amount to nothing, if the symptoms impress upon the observer's mind a perfect image of the disease. In no respect do homœopaths depart so widely from true medical science as in this ignoring of the internal conditions on which the symptoms depend—the crowning glory of the regular profession in our day, being the progress attained, and the accuracy acquired in appreciating pathological changes.

For my own part, I believe the time will arrive when medicine will be justly entitled to rank among the exact sciences; and its progress in that direction already is very great. The eye is explored to its bottom, and alterations of its internal tissues are exhibited by the aid of the opthalmoscope. The laryngoscope penetrates the air passages as far as the vocal chords, and as some say, to the bifurcation of the trachea; the organs within the thorax are thrown open to our thorough inspection, through the agency of auscultation and percussion, so that chest diseases are no longer a mystery to the experienced ear. The uterus and its appendages are examined with as much satisfaction as the fauces; and who can estimate where the investigations of the urine and other secretions, by means of chemical pathology and the microscope, will terminate. Let means be devised for the physical exploration of the head and abdomen, and medicine, disenthralled of theory and speculative reasoning, will stand forth a proud monument of the fidelity of its votaries.

Homœopathy, as shown by a recent writer,* has a considerable infusion of a pseudo-religious element connected with it, and this may explain the adherence to it of certain ministers of the gospel. Having neither reason or facts to sustain it, a belief in its tenets is as much an act of faith as a belief in the incarnation; and that its founder looked upon it in this light, may be inferred from such aphorisms as the following: "It is only by means of the

---

*Prof. J. P. Logan.

spiritual influence of a morbific agent, that our spiritual vital power can be diseased; and in like manner, only by the spiritual operation of medicine, that health can be restored." Now, this is as clear as a sunbeam to some spiritually-minded people, but to such gross poor mortals as the writer, it is stupid, not to say criminal, nonsense.— How truly has it been said: "Man is a dupable animal, quacks in medicine, quacks in religion, and quacks in politics, know this, and act upon that knowledge. There is scarcely any one who may not, like a trout, be taken by tickling."—*Southey.*

After all, I believe there is some philosophy in all systems of quackery, and think some of them may be rationally explained. Hydropathy had its origin during the epoch of our profession, when cold air, and cold water were rigorously excluded from the sick chamber; Preisnitz broke through the restraint, and plunged into the other extreme of employing these elements to the exclusion of all other modes of medication. The rise of homœopathy was due to the immense quantities of medicine given at that period, particularly in Germany; so notorious was this the case in that country, that the Germans now in the United States, judge of the qualifications of a physician by the quantity of medicine he gives them. And Thompsonianism was directly indebted to the outrageous abuse of mercurials and blood-letting; and, in my opinion, the value of these remedies is much more correctly estimated now than formerly. On the whole, therefore, I think the regular profession has been benefitted by each and all of the exclusive systems that have arisen, and that its principles and practice are more nearly correct at this time, than at any former period of its history.

I find that the various methods of physical diagnosis, are diligently applied here in the investigation of disease. Auscultation and percussion are as household words, and practiced to great perfection. The great predominance of pulmonary affections renders these agencies necessary, and invest them with great importance—the proportion of deaths from phthisis, for example, being about one in six. The microscope also is in general use, and yet, strange to say, it receives no attention in the curriculum of the colleges; its utility and the results obtained by it are indeed frequently mentioned, as if to tantalize the student, but his only chance to become conversant with its use and practical application to medicine, is through the agency of private instruction. In view of the invaluable results obtained from the use of this instrument in the examinations of the urine alone, not to mention its application to numerous other products, no medical college should permit its graduates to go forth from its halls, without having imparted to them such knowledge of its use, as will enable them to apply it to chemical medicine.

At the Dispensary clinics on surgery, diseases of the skin, diseases of the chest, and the diseases of children, may be occasionally seen some half a dozen respectable looking females giving strict attention to what may be seen and heard. These are female doctors. The clinics are attended by crowds of medical students, and much of what is said and seen would shock the feelings of a modest woman. A considerable proportion of the diseases presented, are of venereal origin, and are, of course, commented on without any regard to the presence of females. Our profession has been accused of illiberality on account of its opposition to this class of practitioners, and certain organs of public opinion have warmly encouraged female medical education. It sounds nicely, and reads well, to have the sick couch and dying pillow ministered unto by the angelic hand of woman; there is a romance in this kind of thing which is difficult to resist. But it must be borne in mind, that a medical education cannot be acquired by a woman, except at the sacrifice of that modesty which is the charm of her sex, and the most precious attribute of her character. It would be ungallant not to say that I loved the presence and companionship of woman, but I must confess to a deep sense of disgust at the sight of these women. They belong, of course, to the class known as strong-minded, and, without any positive knowledge of the fact, I take them to be old maids. The remedy, in my opinion, for all such vagaries of the female mind, is to impose upon them the fulfillment of the divine injunction, "be fruitful, multiply and replenish the earth." Very truly, yours,

D. C. O'KEEFE.

---

P. S.—A friend has just called my attention to the Philadelphia "Medical and Surgical Reporter" for July 30, which contains an ill-natured criticism on my letter to you, of July 1st. In writing these letters, I think but little, and care less, about their bearing on local interests, and merely give your readers a faithful representation of the impressions I have received. Divested of captiousness, the article of the "Reporter" amounts to this, viz.: 1st. That I deliberately refused to see the greatness of Philadelphia, in Medical Institutions and Medical men, and, 2d, that I foolishly exaggerated the medical advantages of New York. In proof of the first charge, it is alleged that I found but two hospitals in Philadelphia, whilst in New York, I discovered twenty-five; and that I entirely ignored the Pennsylvania hospital. Now, this grossly perverts my meaning, and only shows the grumbling mood into which my "conceited" comparison had thrown the writer. I did not say there were *but* two hospitals in Philadelphia, but that the chief hospital advantages existed in two, which cannot be denied, In like manner, in relation to New York, I stated that two hospitals attract the principal attendance, and only gave a detailed account of an equal number of hospitals in each city. As to ignoring the Pennsylvania hospital, your printer made me say "Philadelphia" instead of Pennsylvania hospital; and the writer in the "Reporter," either displays inexcusable ignorance of his own city, in not seeing that the Pennsylvania hospital was the institution referred to, or, seeing it, he is guilty of duplicity in making it the ground of complaint. The date of its foundation, mode of support, number of patients, fee of attendance, &c., &c., were all given—enough, one would think, to enable the special defender of the "medical emporium," to correct the typographical error. It was of this hospital, and not Blockley, (as the "Reporter" "absurdly" states,) that I said its wards were only accessible to the pupils of the attending physician, and if its vision had not been obscured by prejudice, it would have so understood me. I made no effort to depreciate the greatness of the medical men of Philadelphia—their fame is too well established, at home and abroad, to be affected by my humble opinion. I had the pleasure of meeting some of them—the editors of the "Reporter," among others—and sincerely admire their talents and attainments. Still, my particular wants did not require great men, but good opportunities for observation and self-culture, and this may account for my obtuseness in respect to Philadelphia greatness. To be more explicit. I left home with the intention of spending six months in Philadelphia or New York, and if I entertained any pre-conceived partiality, it was in favor of the former. But as it was of some importance to spend my time as profitably as possible, I determined to examine the facilities at each place, and then make a selection understandingly. With this view, I spent seven days in Philadelphia, not with my eyes cautiously shut, as the "Reporter" has it, but diligently in search of such clinical advantages as I needed. If they existed there, I failed to find them; not but what there are numerous hospital institutions in the city, but it seemed to me so many obstacles and restrictions were thrown in the way, that I left Philadelphia under the serious apprehension, that I should not obtain that full and free access to disease which I desired. Two days in New York exhibited, to my heart's content, a state of things not to be found in Philadelphia in seven. Not alone in extent do they excel, but they are thrown open—literally pressed aud crowded on you.—There is no use, my dear "Reporter," in trying to deny this thing—it is patent to everybody—the way-faring man, though a fool, can see it—it has made itself so palpable, that your own students feel it, spend the summer here, and return to your lectures. Cease, then, to decry the clinical superiority of New York, and to vilify those who maintain it. D. C. O'K.

